# Living with type 2 diabetes mellitus in the Kingdom of Tonga: a qualitative investigation of the barriers and enablers to lifestyle management

**DOI:** 10.1186/s12889-021-11391-7

**Published:** 2021-07-03

**Authors:** Jennifer Taumoepeau, Catherine R. Knight-Agarwal, ‘ Esiteli A. P. Tu’i, Rati Jani, Uchechukwu Levi Osuagwu, David Simmons

**Affiliations:** 1 Faculty of Health, University of Canberra, Canberra, Australia; 2 School of Clinical Sciences, Faculty of Health, University of Canberra, Canberra, Australia; 3 Vaiola Hospital Ministry of Health, Nuku’alofa, Tonga; 4 Diabetes, Obesity and Metabolism Translational Research Unit (DOMTRU), School of Medicine, University of Western Sydney, Campbelltown, Australia

**Keywords:** Type 2 diabetes mellitus, Non-communicable disease, Tonga, Talanoa, Lifestyle management

## Abstract

**Background:**

Despite the increasing prevalence of Type 2 Diabetes Mellitus (T2DM) in the Kingdom of Tonga, little is known of non-communicable disease experiences among adults living in this location. This investigation aimed to explore the barriers and enablers to healthy lifestyle in a group of men and women living with T2DM residing in this Pacific Island nation.

**Methods:**

This qualitative study consisted of three semi-structured focus groups (*n* = 16), conducted at the only Tongan Public Hospital located at Nuku’alofa, capital of Tonga (north coast of the island of Tongatapu). Discussions were audio-recorded, transcribed, cross-checked for consistency, and entered into a word processing document for analysis. Thematic analysis was employed to synthesise results.

**Results:**

Four main themes were identified: (1) Knowledge and Support; (2) Fear and Motivation; 3) Physical and Psychological Environment; and (4) Faith and Culture.

**Conclusions:**

The qualitative findings from this study will assist the future development and information dissemination of culturally appropriate lifestyle-related for men and women living with T2DM in the Kingdom of Tonga. The need for collaboration between practitioners at the hospital, the church, family members, and local traditional healers is important if the lifestyle-related needs and wants of this group of people are to be met.

## Introduction

In 2019, the International Diabetes Federation reported that approximately 463 million people aged between 20 and 79 worldwide were living with diabetes [1]. Of concern, is the increasing prevalence of T2DM in adults from low- and middle-income countries including Kingdom of Tonga [1]. The World Health Organisation developed an approach to monitor any trends and compare information on a regular basis about the risk factors of Noncommunicable disease (NCD) and discuss the status of NCD, strengths, limitations, and future recommendations [2]. The STEPwise approach to surveillance (STEPS) measure three different levels of risk factor assessments [2]. The first step is a questionnaire assessment followed by physical and biochemical measurements [2]. Results gathered from the STEPS survey of 2004, showed that 16.4% of Tongans lived with an elevated fasting blood glucose level (BGL) (BGL ≥7.0 mmol/l: threshold for T2DM) [2–4]. From the period between 1973 and 2012, T2DM prevalence has increased in Tonga from 5.2 to 19.0% (1.9% per 5 years) [3, 4].

Traditionally, like other Pacific islander people, Tongans lived a subsistence lifestyle, producing what they needed to survive with surplus contributing to celebratory occasions [4]. However, significant changes in food habits have occurred in the Kingdom since the end of World War II [5]. There has been a shift away from relatively healthy traditional diets, alongside a decline in physical activity and increased consumption of imported and processed foods, which are typically low in fibre and high in refined carbohydrates and fats [5–9]. This nutrition transition has contributed to a dramatic rise in prevalence of obesity and NCDs such as T2DM, which in turn has led to increased social and economic burden [6, 10].

In a recent systematic review and meta-analysis, the efficacy of lifestyle interventions with emphasis on the management of T2DM and diabetes risk factors in Polynesian people were reviewed [11]. There were eight studies included. Four were randomised controlled trials and the other four were pre-post studies with 1590 participants who met the inclusion criteria [11]. The health outcomes reported in the studies included weight, body mass index, waist circumference, glycated haemoglobin, and blood pressure [11]. Lifestyle interventions were adapted to suit Polynesian people living with and at risk of developing T2DM [11]. In four studies, the findings showed reductions in systolic blood pressure (Weighted Mean Difference, − 9.93 mmHg; 95% Cl, − 10.77 to − 9.09; and *p* < 0.0001) [11]. Across five studies, the lifestyle interventions had no significant effect on weight loss and in two studies, there were no significant reduction in mean HbA1c levels (WMD, − 0.38%; 95% Cl, − 1.15 to 0.39; and *p* = 0.33 [11]. The authors of the review concluded a lack of quality evidence of lifestyle interventions for the prevention and management of diabetes among Polynesian people [11]. They recommended that future programs need to be culturally acceptable and appropriate at all community levels in order to address the growing diabetes epidemic [11].

About three decades ago, the Tongan Medical Association acknowledged the need for specialised T2DM care, with the establishment of a multidisciplinary clinic in the capital, Nuku’alofa, a short time after and the initiation of the National Diabetes Registry in 2004 to monitor diabetes people [12]. Diabetes self-management education is an evidence-based practice that has been found to improve glycaemic control as well as reduce complications. Individuals diagnosed with T2DM in Tonga receive counselling from a qualified Dietitian with assistance from a diabetes nurse [12]. Inpatients on the isolation ward, who are known to be regular users of traditional medicine and have carbuncles or lower limb amputation, are referred for multi-disciplinary diabetes advice [12, 13].

Traditional medicine is used alongside western medicine and is respected by Tongans [12, 13]. Many hold the belief that traditional medicine and prayers are more effective than the hospital, evidence-based treatment [13]. In addition, it is also acknowledged that traditional medicine is woven into the social and religious fabric of a Christian Tonga [12]. Recent qualitative research investigating lifestyle management of T2DM has originated predominantly from developed countries such as Australia [14, 15], Sweden [16], UK, and the USA [17, 18]. Evidence from low to middle-income countries, like those living in the South Pacific, who are significantly affected by T2DM are limited. The aim of this study was to investigate the barriers and enablers to a healthy lifestyle faced by Tongan adults living with T2DM and residing in the Kingdom of Tonga.

## Material and methods

### Setting

The Kingdom of Tonga is a Polynesian country comprised of 176 islands, 36 of which are inhabited. Tonga is the sole constitutional monarchy in the South Pacific [2], made up of four major island groups which are Tongatapu, Ha’apai, Vava’u, and the two Niuas- Niuafo’ou and Niuatoputapu [2]. Nuku’alofa, the capital, is located on the main Island of Tongatapu. The entire country has a population of 100,745 people where 74% of the population resides on the main island [2]. According to the World Health Organisation (WHO), Tonga has the best levels of health in the South Pacific which reflect positively on their primary health care system [2]. All Tongans residing in Tonga have full access to healthcare services with medication accessible free of charge. Nevertheless, NCDs such as T2DM and Cardiovascular disease pose significant challenges for the countries healthcare system [2, 4, 11, 15–18]. Christianity is embedded in Tongan society with health inextricably influenced by this [2, 9]. Food is given and shared among families and church communities with copious amounts being a sign of respect [2]. Faith is a motivating factor for the maintenance of healthy lifestyles for some Tongans [12]. However, this can have a negative effect on health where an individual may live a poor lifestyle and hold the belief that God will assist in the healing process if disease occurs.

### Design

The study was conducted at the largest public health facility in Tonga, Vaiola Hospital with focus group discussions undertaken in one of the allied health buildings. This qualitative research was used to gain an understanding of participants underlying beliefs, motivations, actions, and thoughts. Such research considers reality to be socially constructed and aims to produce subjective findings through a process of inductive reasoning [19]. With this in mind, *Talanoa (the act of telling a story)* was observed during the course of the research process [20, 21]. The term is derived from two Tongan words: *tala*, to talk or tell, and *noa*, anything or nothing in particular. Together, *talanoa* means that the *noa* enables listening to and learning from the *tala*, without the burden of a calculated agenda [20]. Talanoa incorporates focus group discussions in a way that is culturally appropriate for Tongans. Talanoa is how Tongans verbally communicate with each other and the traditional way of sharing knowledge, history and making connections with family and friends. The process emphasises the need to tell stories without the camouflage of what is, and what is not, important [20, 21].

The Tongan language is functional and unique in the way that it links metaphors, cultural values, beliefs, and poetry to bring out knowledge and information [20, 21]. Therefore, translations may not be simple when explaining something from a western perspective [20]. In Tonga, therefore the only true way to gain information is through native tongue expression [20, 21].

### Participants

This study utilised purposive sampling which requires that individuals are deliberately selected with an explicit purpose in mind namely to address the research aim and because they are rich sources of data in relation to this. Information about the study was disseminated to men and women living with diabetes by health professionals from the T2DM multidisciplinary clinic. A short while after, men and women attending the clinic were approached by a student researcher. For those individuals who expressed interest in participating in the study, a flyer was provided which contained information about the aims and objectives of the research, ethics approvals, and the consent process.

Participants were included if they were residing in Tonga (both male and female), of Tongan ethnicity, aged ≥18 yrs., and had been formally diagnosed with T2DM. Men and women who met the study criteria and agreed to participate provided signed consent and were given a unique identifying number to ensure anonymity.

### Data collection

A semi-structured interview guide was developed by the research team based upon a review of the published literature [1, 7–9, 11, 21, 22]. The majority of questions were kept deliberately open providing cues for participants to talk with a minimum of interruption and without judgment. Three focus groups, lasting approximately 1 h each, were conducted in May 2019. The facilitator, of Tongan heritage, conducted the focus group discussions in her native tongue as a way to aid authentic *talanoa* [20]. Each focus group discussion was audio-recorded and transcribed verbatim, translated into English, and then entered into a word processing document for analysis. The saturation of data was determined after the conclusion of the third focus group. Focus group participants were chosen from the list of patients attending the Diabetes clinic and demographic data such as occupation, age, gender height, and weight as well as glucose levels.

### Data analysis

Thematic analysis techniques, inspired by Braun and Clarke, were followed [22]. Transcripts were coded in detail to single out recurring patterns and quotes were given a unique qualifier to identify each participant. This process was undertaken iteratively in addition to ongoing discussion between all researchers. It also served as an important means of triangulation resulting in the final set of superordinate themes [19].

## Results

Sixteen adults (56% women and 44% men; mean age 50.7 ± 27.1) with T2DM took part in the study. The first focus group consisted of three females and three males, the second and third focus group consisted of two females and three males each. Four superordinate themes were determined.

### Theme 1 - knowledge and support

Despite the prevalence of T2DM in Tonga, some participants reported having very little knowledge about its prior to diagnosis with one participant reporting that: “*In 2007 I got diagnosed … ... I felt like a knife stabbed me right in the heart. My blood sugar levels went up to 32.9 where I got rushed to the hospital at that time. I didn’t know what was going on around me or what hyperglycaemia or hypoglycaemia was, all I remember was feeling sleepy and I must have passed out”* (Pauline FG1). Others reported that they were aware of the hereditary component of the disease: *“I have cousins and a sister with diabetes, so I had a feeling that’s why I have it”* (Puafisi FG3).

Participants were cognizant that food intake (and the nutrient contained in these foods) affected their glycaemic control: *“I have had sugar since 2000, up until now I haven’t had hypo or hyper incidents such as what the others are talking about. I can control my sugar intake and I am very active physically and I can resist sugar in my tea and coffee. The highest my BGL’s have gotten to is 8”* (Maleta, FG2). Another reported: *“I have cut drinking anything sweet …… I started drinking lime (and water instead) … … I can see an improvement as before I used to (go) …… to the toilet 3 times during the night but now I only go once”* (Uaisele FG2). Likewise, exercise was perceived by some participants as an important way to keep BGL’s in check: *“I am more active nowadays which isn’t quite what I used to do back then (before I was diagnosed)*” (Nai, FG1).

In addition, there was recognition that the dietary knowledge acquired for optimal BGL control had the potential to extend beyond individual health promotion as one participant exclaimed*: “I am more careful now and I am thinking about the health sides of foods I prepare for my family”* (Ata, FG1).

Knowledge is even more effective in combination with support, which in Tonga, comes primarily from the immediate and extended household. Family is at the very core of society with one participant sharing her belief: *“I think I would be dead by now if it weren’t for my family”* (Sione, FG2). Some found the transition from diagnosis to living with the disease relatively easy: *“because my husband has diabetes, we as a family eat healthy and exercise to support him …… so, it feels better doing things together”* (Losaline, FG3). Conversely, for others it had been a rocky road and … … *“challenging …… to live with diabetes”*. (Ata, FG1).

Participants acknowledged the supportive expertise available at the specialist diabetes clinic: “*my diabetes is uncontrollable which Is why I come to the hospital … … I get advice on how much food and when to eat and what not to eat”* (Mele, FG1). Another participant expressed similar sentiments: “*I think it is very important to get an in-depth understanding to what contributes to getting diabetes (so you then know how to manage it)” (*Loleini FG2).

Despite the support systems available and the knowledge that poorly controlled diabetes could lead to adverse health outcomes, some participants were still complacent: “*I am going to die of something one day so might as well eat and drink to my heart’s content*” (Saimone, FG1). There are limited resources that can help raise awareness on Diabetes and the importance of self-management and access to glucometers in Tonga which contributes to poorly controlled diabetes.

### Theme 2 - fear and motivation

For many, being diagnosed with T2DM felt like a death sentence with one participant reporting: *“I felt so sad. I didn’t feel like living any longer, I couldn’t eat or sleep because I knew that I’d have to take tablets for the rest of my life”* (Ata, FG1). Participants considered amputation a major consequence of T2DM and several were fearful that this may be their future reality: *“I’m always scared that I might get injured especially when most people here in Tonga with diabetes, if they’re not careful with managing their diabetes and doing what the doctor says have their lower limbs … … amputated”* (Ata, FG1). The anxiety of losing mobility was an incentive to maintain a healthy lifestyle for some with one participant stating: “*My biggest fear is lower limb amputation so that is a great motivator for me and management of my diabetes”.* (Maleta, FG2). For others leading a healthy lifestyle was a reason not to be fearful of complications: “*For 15 years I have had Diabetes ……*. *after I retired, I was diagnosed. I maintain my sugar levels, so I am not worried or feel scared”* (Nai, FG1). Other complications were discussed within the group as inducement to follow a healthy lifestyle: *“I am 60 years of age … … I am going blind in one eye and I was told that … … the other side will eventually get worse if I don’t look after my sugar levels”* (Mele, FG1). Some participants appeared to be intrinsically motivated with one man claiming: *“I had no problem at all from the day they told me (I had diabetes) and the foods that I should not eat”*. (Nai FG1). Others were motivated to follow a healthy lifestyle by extrinsic forces: *“There are days where I feel so hungry, I could eat mutton flaps (laughs) … … while I drink kava you know? But I want to live to see my grandchildren grow old*” (Saimone, FG1). Arcia Tecun and associates examined the history of the Tongan Kava that originated in Tonga in the island of ‘Eueiki [23, 24]. The root of the plant is pounded and mixed with water to produce a muddy brown drink [23, 24]. It is the preferred beverage among men in Tonga and known as an alternative for alcohol. Kava consumption is common practice and is significant in the Tongan culture [23, 24]. Kava is used in cultural ceremonies, funerals, birthdays, weddings, and other social gatherings as a way of bringing together family and friends, making connections and building relationships and allowing people to interact with one another and share experiences through talanoa [23, 24].

Tongans are renowned for their love of gathering, preparing and consuming food. For many, mealtimes are the most pleasurable activity of the day as one participant exclaimed: “*There are times where I can’t control my eating (laughs) … … and my BGLs are uncontrolled”* (Nia, FG3). It was acknowledged that the motivation to eat healthily can be difficult: “*it is hard for me to eat the right foods when I am hungry (laughs) or eating less is hard when something is so delicious (laughs)”* (Mafi, FG3). Another participant made the comment: *“I have a weakness for … … sugary drinks and … … the doctor at the Suka clinic would always tell me off (laughs) because when my sugar levels rise, he knows it’s because I’ve drunk something sweet”* (Uaisele, FG2). Participants confessed that household income often affects both motivation and purchasing power: *“my family doesn’t like the foods I eat so it is extra expense. Most of the time I will just eat what they will eat*” and “*healthy foods will (only) be consumed on days we can have it ……*” (Kalo, FG3).

### Theme 3 – physical and psychological environment

Food environments are influenced by the systems which supply them, and vice versa. Many participants, mostly urban dwellers, admitted to being reliant on local markets for their groceries with processed foods often being cheaper than fresh produce. Easy access to low cost processed foods was a barrier to healthy eating for several as one participant exclaimed: *“Turkey tails … … hot dogs … I cook them with taro and throw in whatever … … that is cheap”* (Mele, FG1).

However, a few participants had access to agricultural land enabling them to tend their own patch: “*God gives us (soil) to make use of and grow our own vegetables”* (Losaline, FG3) and “*I go to the bush and grow crops*” (Nai, FG1). Likewise, an individual’s workplace environment can influence not only access to food, but the type and quantity consumed as one participant commented: *“I am working at a restaurant and I am exposed to food on a daily basis. I usually eat while I’m at work and I don’t care about my sugar levels and my diabetes … … every time I finish work, I feel very drained (and) sometimes lose feeling in my hands. When I test myself at home with my glucometer my sugar levels would increase to 13 due to what I have eaten that day”* (Sapoi, FG2). Some participants intimated that their work routines did not support strategies to maintain good glycaemic control such as undertaking regular physical activity: *“I don’t have time to exercise because I leave home early and don’t get home till late at night”* (Kalo, FG3). Others recognised that home duties are an important form of energy expenditure: *“I always do gardening early in the morning every day and mow the lawn as well, so I try and find any work … … to keep moving”.* (Uaisele FG2, L12). Very few participants claimed to have access to their own glucometers making daily ‘*suka*’ monitoring near impossible. Nevertheless, a fortunate few were in possession of their own: “*I have a glucometer which is so useful for me to be able to use it at home”* (Uaisele FG2).

### Theme 4 – faith and culture

Faith and culture are inextricably connected to the environment with which Tongans live. The Christian God, creator and preserver of all things, is at the core of how most individuals manage T2DM with one participant stating: *“Asking the man upstairs …… to help us get through because he always helps …… that’s why we pray (laughs)”* (Uaisele FG2) and another claiming*: “That’s the main thing having faith but working hard knowing God will do the rest” … …* (Kalo, FG3). For many, faith was an unwavering source of hope and a type of “spiritual medicine” which contributed to the daily management of their condition as one participant exclaimed: *“I have uncontrollable diabetes with complications in my eyes, kidney and … … we should not let diabetes dictate the way we live. That is why we pray and ask God for guidance and to help us keep going because, He is the only one that can help us with our health journey, and He always gives us enough that we can handle (smiles)”* (Loleini, FG2). Christianity is embedded in the Tongan culture and people believe that having faith in God and praying daily will help them live a healthy lifestyle.

Cultural environments shape the way that individuals develop their core ideologies. Traditional medicine is a fundamental part of Tongan ideology as one participant reported: *“My grandparents and parents used a traditional medicine that balances sugar levels …. It worked for them, now it works for me”.* (Soakai, FG2). And another made the comment: “*Ango (Zingiberaceae tubers) is used in my family for upper respiratory infection … … it works for my diabetes (as well)”* (Maleta, FG2). For other participants, taking prescribed ‘modern’ medication was not an option: *“I started taking metformin, but I don’t really like taking metformin …. it makes me feel very weak and sometimes I would feel a little numb so ……. I am not taking the pills”* (Sapoi FG2, L13). This participant’s faith in traditional medicine was unwavering as he went on to say: *“I also try the Tongan medicine that helps lower my sugar levels, the angoango plant, and it is said to cure diabetes”* (Sapoi FG2, L13).

## Discussion

This qualitative study explored lifestyle-related factors influencing T2DM management among Tongan adults residing in the Kingdom of Tonga. Participants in this study had very little knowledge of the aetiology of T2DM prior to their diagnosis, a finding that has been reported previously [4, 11, 12, 25–27]. The participants acknowledged receipt of education through the hospital’s specialist clinic, and how important this was, once they knew they had the condition. Likewise, a recent qualitative study by Dearie and colleagues explored the knowledge and attitude towards diabetes among i-Taukei Fijians living in Australia [28]. Participants generally demonstrated an understanding of the mechanisms involved in the development of T2DM, but some were unable to define the condition or believed there was a general lack of knowledge around diabetes in their community [28, 29]. A retrospective audit of the Tongan National Diabetes Registry reported that only around 3% of individuals living with diabetes during the period 2011 to 2012 accessed health care, indicating that many Tongans failed to utilise relevant services [12].

It has been well established that diabetes complications can be reduced by implementing positive lifestyle changes [11, 25, 28, 30–32]. Participants in our study spoke about their fear of limb amputation recognising that poor glycaemic control may lead to this outcome. Preventing foot ulcers is an important strategy for avoiding amputation. Complication awareness should be a key area of focus in any lifestyle intervention that aims to promote optimal glycaemic control in people living with T2DM. The Tongan Ministry of Health’s NCD Strategy 2015–2020 has four key areas of action including alcohol reduction, good nutrition, healthy environments, and tobacco control. Interventions, which focus on one or more of these areas, are carried out in schools, churches, and various workplaces with the aim to reduce NCDs morbidity and mortality [25, 26]. However, despite this strategy there are minimal resources available to support this work.

The Tongan Ministry of Health has collaborated with The Ministry of Education, the Health Promotion Foundation, the WHO, and the University of Otago to develop a School Food Policy- A Pathway to a Healthier Life [25, 26, 33]. In 2007, this food policy came into effect and was updated in 2012, prior to its launching on the 12th of November 2019 [25, 26, 33, 34]. The policy aims to ensure that healthy and nutritious food is readily available to students both at home and in schools [25, 26, 33]. By developing good habits and increasing knowledge regarding what a healthy diet consists of in early life, prevention of T2DM may become more of a reality.

Food is of cultural importance in Tonga posing a challenge for healthcare interventions. Some participants in our study acknowledged that a barrier to maintaining optimal BGL’s was their propensity for high fat, sugary foods, and limited ability to purchase healthy options (due to either cost or availability) [11, 34]. A 1998 study aimed to identify and quantify major hurdles to diabetes care from a multiethnic, urban community perspective (mainly New Zealand Europeans, Maori, and Pacific Islanders) [35]. Barriers to care were quantified among 1862 (2.1%) individuals living with diabetes out of a total surveyed population of 90,477 [35]. In spite of major cultural differences between participants, the top 10 barriers were similar between the ethnic groups [35]. The most important barriers perceived included that benefits of self-care were outweighed by the disadvantages (20% Europeans, 20% Maori, 29% Pacific Islanders, 16% others, *p* = 0.001), lack of community-based services (13% Europeans, 27% Maori, 25% Pacific Islanders, 11% others, *p* = 0.001) and the limited range of services were available (15% Europeans, 22% Maori, 20% Pacific Islanders, 14% others, *p* = 0.05) [35]. The authors reported that systematic action to reduce the impact of these barriers, in both patients and populations, could result in an improvement in diabetes outcomes [35].

Foods considered too fatty for consumption by “Western” standards, such as lamb flaps, are enjoyed in Tonga as delicacies, and eating large portions of food is considered polite, especially in the context of social gatherings [8]. A recent review by Underhill and Singh-Peterson reported that purchasing fresh fruit and vegetables in Tonga can be a highly capricious venture [33]. For much of the year, commercial fruit and vegetable supply chains are restricted to a few retail outlets selling limited quantities of both local and imported produce [33]. Similarly, Morgan and associates conducted focus group discussions with both indigenous and Indian Fijians residing in Suva to investigate factors influencing fruit and vegetable intake [29]. They found that increasing preferences for processed and imported foods, and inconsistent availability and affordability of high-quality, low-priced, fresh produce, were barriers to fruit and vegetable consumption [29]. Promoting dietary habits based on increased consumption of fresh fruit and vegetables should be a critical first step in tackling NCD’s.

Nevertheless, healthy food accessibility is an issue in Tonga. Corner shops have cheaper alternatives to bottled water and minimally processed food items such as sugary drinks and products high in fat and simple carbohydrates are abundantly available [4, 8, 10]. In Tonga, there are small corner shops in every village where Tongans buy their weekly groceries. Policies need to focus on increasing the availability of affordable, nutritious products and decreasing the number of cheap, highly processed food imports [12, 25].

Evidence for the positive influence of ‘family’ on an individual’s glycaemic control has been accruing [11, 28, 29, 36, 37]. In the USA, McElfish and colleagues’ pilot-tested a home-based model of diabetes education with Marshallese individuals and their extended family members [38]. More than three-fourths (78%) of participants were retained in the study and a 7% reduction in HbA1c was observed among those with T2DM [38]. The authors concluded that culturally targeted diabetes interventions, which embrace family members, may lead to better results for individuals with T2DM than traditional self-management education programs [38]. Similarly, a recent study from the USA illustrates how leveraging spiritual beliefs in an African American church-based diabetes intervention may promote positive behaviour change [39]. Pacific Island churches play a significant role in the culture and authoritative systems of the communities they serve, as well as providing a place for fellowship and communication [40–42]. A recent prospective, a pre-post study of church-wide diabetes education and support programme was carried out with a group of Samoans living in Sydney, Australia (40). Overall, 68/107(63.5%) of participants completed before and after intervention measures (mean age 48.9 ± 14.2 years; 57.2% female) [40]. The primary outcome, HbA1c, dropped significantly between baseline and follow-up among participants with known diabetes (8.1 ± 2.4% (65 mmol/mol) vs 7.4 ± 1.8% (57 mmol/mol); *p* = 0.040) [40]. Church-based programs may be more realistic when aiming for adherence to healthier lifestyle behaviours as this approach has been shown to be effective in improving diabetes complications as well as diabetes management in Pacific Island populations elsewhere [40–42].

A recently published review examined the importance of incorporating traditional herbal medicines into Fiji’s modern health care system [43]. The authors identified a number of physiological, spiritual, and social advantages [43]. Understanding the role of both western clinicians and local traditional healer’s practices is necessary as efficacy is clearly interwoven with culture, faith, and politics [43]. Further evidence-based research is needed in this area. Lifestyle-related interventions that include the church, family members plus the input of local healers may contribute to not only a greater understanding of why Tongans choose to follow certain treatment pathways but ultimately lead to better physical and psycho-social outcomes for people living with T2DM.

## Conclusion

In summary, the present study showed that men and women with T2DM living in Tonga had very little knowledge of the aetiology and management of T2DM prior to their diagnosis, but acknowledged that education provided through the hospital’s specialist clinic was important after they have been diagnosed. The findings from this study may assist the future development and dissemination of lifestyle-related information for men and women living with T2DM in Tonga. Collaboration between practitioners at the hospital, the church, family members, and local traditional healers is paramount if the lifestyle-related needs and wants of Tongan adults living with T2DM are to be met. Extra resources in terms of staffing, specialised training, and funding are required to achieve this.

## Data Availability

Data are however available from the authors upon reasonable request and with permission of Jennifer Taumoepeau.
